# Transition from specialist to primary diabetes care: A qualitative study of perspectives of primary care physicians

**DOI:** 10.1186/1471-2296-10-39

**Published:** 2009-06-06

**Authors:** Sharon Brez, Margo Rowan, Janine Malcolm, Sheryl Izzi, Julie Maranger, Clare Liddy, Erin Keely, Teik Chye Ooi

**Affiliations:** 1 Foustanellas Endocrine and Diabetes Centre, The Ottawa Hospital, 4th Floor Riverside Campus, 1967 Riverside Drive, Ottawa Ontario, K1H 7W9, Canada; 2 Department of Family Medicine, University of Ottawa, C.T. Lamont Primary Health Care Research Centre, Elizabeth Bruyere Research Institute, 43 Bruyere St Ottawa Ontario, K1N 5C8, Canada; 3 Division of Endocrinology and Metabolism, University of Ottawa, 4th Floor, 1967 Riverside Drive, Ottawa, Ontario, K1H 7W9, Canada

## Abstract

**Background:**

The growing prevalence of diabetes and heightened awareness of the benefits of early and intensive disease management have increased service demands and expectations not only of primary care physicians but also of diabetes specialists. While research has addressed issues related to referral into specialist care, much less has been published about the transition from diabetes specialists back to primary care. Understanding the concerns of family physicians related to discharge of diabetes care from specialist centers can support the development of strategies that facilitate this transition and result in broader access to limited specialist services. This study was undertaken to explore primary care physician (PCP) perspectives and concerns related to reassuming responsibility for diabetes care after referral to a specialized diabetes center.

**Methods:**

Qualitative data were collected through three focus groups. Sessions were audio-taped and transcribed verbatim. Data were coded and sorted with themes identified using a constant comparison method. The study was undertaken through the regional academic referral center for adult diabetes care in Ottawa, Canada. Participants included 22 primary care physicians representing a variety of referral frequencies, practice types and settings.

**Results:**

Participants described facilitators and barriers to successful transition of diabetes care at the provider, patient and systems level. Major facilitators included clear communication of a detailed, structured plan of care, ongoing access to specialist services for advice or re-referral, continuing education and mentoring for PCPs. Identified provider barriers were gaps in PCP knowledge and confidence related to diabetes treatment, excessive workload and competing time demands. Systems deterrents included reimbursement policies for health professionals and inadequate funding for diabetes medications and supplies. At the PCP-patient interface, insufficient patient confidence or trust in PCP's ability to manage diabetes, poor motivation and "non-compliance" emerged as potential patient barriers to transition. Incongruence between PCP attitudes and expectations related to diabetes self-management and those of patients who had attended a multidisciplinary specialist center was also observed.

**Conclusion:**

This study underlines the breadth of PCP concerns related to transition of diabetes care and the importance of this topic to them. While tools that promote timely information flow and care planning are cornerstones to successful transition, and may be sufficient for some practitioners, appropriately resourced decision support and education strategies should also be available to enhance PCP capacity and readiness to resume diabetes care after referral to a specialist center. Characteristics of the patient-care provider relationship that impact discharge were identified and are worthy of further research.

## Background

In Canada, as in many parts of the world, Type 2 diabetes (T2D) prevalence is increasing at unprecedented rates. Between 1995–2005 in Ontario, Canada, the number of adults diagnosed with T2D rose by 69% [1]. Forecasts indicate that this rate of growth will be surpassed over the next decade [1]. While primary care physicians (PCPs) provide the majority of diabetes care in Canada, they are at times, challenged in their efforts to fully meet the needs of their patients with diabetes as the disease progresses or when self-management demands become complex [2]. PCPs refer to specialists not only when their threshold for comfort with diabetes management is surpassed, but also in response to consumer demand. Younger adults, representing the fastest growing group with diabetes in Ontario, have demonstrated a preference for direct and rapid access to specialist care, aggressive treatment and more involvement in health care decision making than older adults [3,4]. Inability to gain timely access to specialized diabetes consultation teams may contribute to PCP and patient frustration and sub-optimal disease management [5,6].

Diabetes specialists also experience the pressures associated with the diabetes epidemic. The involvement of specialist teams in the provision of diabetes care is common in large Canadian, urban communities where multidisciplinary diabetes services are more readily available [7]. For example, in Ottawa, the Canadian national capital and site for this study, approximately 29% of people with type 2 diabetes receive all or part of their diabetes care from specialists [7]. This referral rate is remarkably similar to other large urban centers in the Province [8].

Since primary care providers are the gatekeepers to specialist referral in Canada, understanding their perspective is important to the selection, development and application of strategies that will facilitate not only access to specialist diabetes care but also transition *from *specialist care. Previous research has identified factors influencing PCP decisions to refer to specialists and the nature of the specialist-PCP relationship [6,9-11]. Characteristics of effective communication between specialists and PCPs have also been studied [12,13]. On the other hand, very little has been published on the reverse process of discharge from diabetes specialist clinics. We recently published a survey of 177 PCPs that indicated patient adherence and access to diabetes nurse educators were significant barriers to transition from specialist care, while structured consult and discharge letters were identified as important facilitators of successful transition [14]. A United Kingdom study group, addressing this topic, found that primary care providers were willing to resume care for up to 48% of patients referred to specialist services [11]. This group also identified barriers to discharge from the specialist perspective [15,16].

In response to this literature gap, and a need to gain a more in-depth understanding of the determinants of successful transition of care from the perspective of PCPs, we undertook an exploratory, qualitative study to address the following question: What do primary care physicians think are the barriers and facilitators to transition of patients from specialized diabetes care back to primary care?

## Methods

### Design

We selected a qualitative, focus group methodology for this research, as it is an effective way to use group interactions to explore understudied, novel, or complex ideas, perceptions or experiences such as diabetes transitional care [17-20].

### Setting

This study was carried out through the Foustanellas Endocrine and Diabetes Center (FEDC) of The Ottawa Hospital, the regional, academic referral center for adult diabetes care in Ottawa, Ontario in partnership with members of the Department of Family Medicine at the University of Ottawa. The diabetes center serves a catchment area of over one million people and it has approximately 20,000 diabetes related patient visits registered each year. An interdisciplinary team of endocrinologists, diabetes nurse educators, dietitians, chiropodists and social workers provide care through on site and tele-health clinics, individual appointments, and by telephone. A variety of individual and group self-management education programs are also available. In Ontario, a PCP referral is required to access a diabetes specialist, but the choice of consultant, timing and reason for consultation are at the discretion of the individual referring physician. Fees associated with specialist referrals are covered by a universal health insurance plan.

### Participants

We purposefully selected potential participants from a database of PCPs who had referred patients to the FEDC during an 18-month period between September 2004 and February 2006. To ensure representation based on referral frequency, we generated lists of PCP's who referred less often (1–3 patients in the 18 months) as well as those who referred more frequently (4–9 patients in 18 months). These two groups accounted for 96% of PCP referrals. We invited every tenth PCP on each list to participate. As a result, a variety of referral frequencies, practice types (solo, group) and settings (rural, suburban and urban), were represented in our sample.

Potential participants were contacted by telephone. The study was introduced and if interest was expressed, we determined the individual's eligibility for inclusion. Eligibility criteria included having referred patients with diabetes to the FEDC, being a family physician or general practitioner, and having five or more years of experience in family practice. It was made clear that participation was voluntary and that we would provide a small honorarium.

Although two focus groups of 7–8 individuals were originally planned, we conducted a third one to help verify themes and findings from the initial sets. Substantial content saturation was reached at the end of session three as indicated by recurrent identification of common themes and emergence of limited new information. A total of 26 PCPs agreed to participate in this study. Recruitment was terminated once the desired sample size was obtained for each focus group.

### Procedures

This study was reviewed and approved by the Ottawa Hospital Research Institute Ethics Board as protocol #2005434-01H, on March 7, 2006. Following ethics board approval, focus groups were held using standardized procedures [19,20]. PCP's provided written informed consent prior to participating. A moderator's guide was used to maintain consistency of methods across groups. Discussion questions addressed primary care physicians' expectations of specialist care and perceived barriers, facilitators and concerns related to the transition of diabetes care. For the purpose of the study, "transition" was described to participants as a supported discharge of responsibility for diabetes care from specialist to PCP. After this semi-structured discussion, participants were asked to rate the usefulness of a variety of transition support strategies that had been identified a priori from a review of the literature and a survey of PCPs [14]. Participants were invited to add strategies from their own experience to this list. The group was then encouraged to discuss the application and key characteristics of the tools or strategies they had prioritized as being "most useful" to transition.

Discussions lasted approximately two hours and were led by 2 facilitators. The co-facilitators were a University-based researcher with expertise in qualitative methods and health services evaluation (MR) and an endocrinologist (JM) who was not previously known to most participants. All focus groups were audio taped and transcribed verbatim. An independent observer took notes as a back up to the taping and to capture non-verbal feedback. At the end of each focus group there was a debriefing session between the observer and the co-facilitators in which divergent findings, commonalities and differences between participants and among the groups were identified and discussed.

### Data Analysis

Two researchers (MR, SB) were involved in coding the data. One reviewed the transcripts and used an open and axial coding style and a constant comparative method of analysis to identify words or phrases that stood out as potentially significant [20]. In-vivo codes were used as much as possible to label categories using the words or phrases of the participants [20]. These terms were developed into categories or themes to capture the qualitative information and to create a coding manual. The second researcher, an advanced practice diabetes nurse educator, reviewed the coding independently. Inconsistencies were solved through discussing the meaning of a code and reaching consensus.

To enhance the trustworthiness of the data, sessions were audio taped and transcribed, disconfirming evidence was consciously searched and thick descriptions were provided of participants' thoughts and feelings via quotations and examples to confirm themes and patterns.

## Results

### Participant Characteristics

Of the 26 physicians whose attendance was confirmed, 22 (85%) participated in the focus groups, eight in the first and seven each in the second and third groups. Unexpected scheduling conflict was the reason given by all of the non-participants. Table 1 presents participant characteristics. Based on self reported data, a typical participant had approximately 20 years in practice, worked in a group setting in an urban location. There was significant heterogeneity across participants in respect to proportion of patients in each practice with diabetes, the proportion of cases identified by the PCP as "complex", and the rate of referral to a diabetes specialty clinic.

**Table 1:** Characteristics of participants (N = 22).

**Item**	**Median**	**Mean (or %)**	**SD**	**Min**.	**Max**.
Years in practice	20.0	20.6	8.3	7.0	38.0

Type of practice	--	Group = 68.2%	--	--	--
		Solo = 31.8%			
		Academic = 4.5%			

Location of practice	--	Urban = 85.7%	--	--	--
		Suburban = 9.5%			
		Rural = 4.8%			

Percentage of patients in practice with diabetes	10.0	13.3	8.3	2.5	40.0

Percentage of patients with diabetes having complex treatment or self care needs	25.0	33.7	29.2	3.0	100.0

Percentage of patients with diabetes referred to the FEDC by PCP	12.5	24.1	25.1	0*	80.0

*PCP's patient referred directly to FEDC through hospital admission rather than PCP request

### Focus group themes

Themes related to successful transition of care emerged from participant discussion and were clustered around three foci: provider's readiness for transition, the patient's readiness for discharge, and current health care systems factors (Table 2).

**Table 2:** Themes related to successful transition identified by primary care physicians

**Primary care physician readiness for transition of care from specialist**
• Degree to which PCP expectations of specialist referral have been met-"*support received for complex patients*", patient has had "*access to specialized multidisciplinary team*"
• PCP perceptions of "*time*" and "*workload*" associated with diabetes care
• PCP "*knowledge*" and "*confidence*" related to medication adjustment and behaviour change
• Alignment of PCP expectations and "*attitudes" *with those of patient/specialist referral center.

**Patient readiness for discharge**
• "*Self management*" abilities, "*complianc*e", attitude about "*seriousness of diabetes"*
• *"Ongoing access to education" *and resources
• Level of patient " *trust*" in primary care provider, strength of relationship with specialist team
• Degree of alignment of "*patient self management expectations" *and treatment goals with PCP/specialist center.

**Systems factors and transition of care from specialist**
• Use of "*effective communication, coordination of care"*, "*individualized care plans"*, "*ongoing phone advice", "diabetes passport"*.
• Ease of "*access to support*" services, timely re-referral for patients and physicians
• *"High costs*" of diabetes medications and supplies.

### Primary care physician readiness for transition

Four themes directly related to the PCP were associated with successful patient transition from a specialist center. These included the degree to which the PCP's expectations of the specialist referral were met; perceived time associated with providing diabetes care and PCP workload; PCP knowledge and confidence related to diabetes therapy; and whether or not his or her expectations and attitudes were aligned with those of the patient and specialist center.

PCP readiness for transition of diabetes care was dependent upon whether or not their expectations of the referral and assumptions about the role of the referral center were met. Considerable variability in reasons for specialist referral was noted; however, all focus groups identified access to a specialized multidisciplinary team (endocrinologists, nurses and dietitians), and expert assistance with complex cases as expectations of referral to the diabetes center. One participant indicated that specialist referral centers *"should accept even the simple case"* (FG1). Complex patients were generally described in two ways, those who had *"difficult"* temperaments or who were *"non-compliant"* with therapy, and those individuals with multiple co-morbidities or who required intensive medication regimens including combinations of oral medications or insulin. Transition of diabetes care was not expected by PCPs for all patients referred to the specialist team. The benefits of indefinite specialist care were identified for those who were younger at diagnosis *"in their 20s"* (FG 2 & 3), those needing *"ongoing team care"* (FG 1 & 2), and those with *"multiple complications"* (FG 2 & 3). While some referring PCPs expected comprehensive, long term care by the specialist center, others hoped to receive specific services or targeted interventions such as treatment options advice or focused self management education: *" [It] would be a wonderful expectation; to be able to say I need the full service or I only need part of the service"* (FG1).

Insufficient time to adequately address diabetes care needs in general practice was seen as a significant barrier to successful discharge. *"Sometimes I feel frustrated because I know that I should really give more time to that patient, especially patients with complications...consciously I need to feel good about myself as a doctor and not to give the time needed to that patient really gives me part of my daily frustration because again am I going to spend 15 minutes or 45 minutes with the patient? ...that's a source of stress, you know, especially with patients with multiple facets like diabetes" *(FG1).

Participants identified gaps in their own knowledge and confidence related to current treatment of diabetes, particularly the need for updated information and experience with insulin therapy, as an impediment to transition: *"One of the problems I can see is that I watch residents (medical trainees) in the hospital and they are pretty good with insulin, and pretty comfortable with it, but once you get out into a community the size of Ottawa where you're referring here (to specialists) to get your patients started on insulin, then the GP is losing his knowledge very quickly, because he is not ordering the insulin... you know if you are not doing something every day you become rusty fairly quickly and then you become insecure (FG 3)*. While there was a high level of awareness of evidence-based treatment guidelines, opportunities for ongoing applied learning or mentoring with members of the specialist diabetes team were identified as being supportive to effective transition of care.

Finally, when patient, PCP, and specialist expectations or understandings of elements of diabetes management were not aligned, participants felt successful discharge could be negatively affected. Identified areas of potential disagreement included incompatible understandings of blood glucose targets, diabetes self-management behaviors, or responsibility for treatment decision-making.

### Patient readiness for discharge

Patient readiness for discharge was consistently associated with patient behaviours and attitudes and much less frequently identified as being dependant upon attainment of clinical endpoints such as optimized glycemic control, or meeting lipid and blood pressure targets. The ongoing ability of patients to adopt and maintain self-management behaviours such as following a recommended diet, independently adjusting medications (especially insulin) or knowing how to "*deal with sick days*" were identified as facilitators to discharge. PCPs noted that attendance at specialist clinics helped patients *"own their disease" (FG 2)*. This was described as patients taking greater responsibility for self monitoring "...*they take charge and understand what is a good acceptable (blood glucose) level, what is a hypoglycemic level, when should they be going to the emergency room when something isn't quite right..." *(FG3). Using tools that support self-management and accountability was seen as a facilitator of transition. A diabetes passport, maintained and carried by the patient to record the treatment plan and track progress towards desired outcomes, was identified as a very useful means of supporting ongoing patient involvement in care, "*it could help the patient feel kind of active in his own care *..." (FG2).

Participants noted that transition back to PCP might be impeded by patient "*distrust*" or lack of confidence in the family physician's judgment about diabetes management, *"...patients are sometimes stubborn, and they don't want to hear it from us, they'd rather hear it from an endocrinologist than hear it from us... " *(FG2). *"The fact that they are seeing you people (specialists), they realize they have a serious disease now" *(FG1). PCPs looked to specialists as allies who could reaffirm to patients the ability of primary care providers to effectively care for diabetes after discharge. When patients have developed a trusting relationship with the specialist team, or have a strong preference to continue to receive diabetes care from the specialist center, participants perceived that discharge could become more difficult: *"...if they've been seeing an endocrinologist every three months, for five years, they end up with a doctor-patient relationship that they want to continue. So the longer you (specialists) hang on to them, the harder it is to transfer back to primary care" *(FG2).

Misalignment of patient and PCP attitudes and expectations related to self-care behaviours, treatment targets, and responsibility for diabetes care, emerged as potential barriers to successful transition. The term "*non-compliance*" was used by participants in all of the focus groups to describe situations when patients did not behave as expected by the PCPs. For some participants, "*better compliance" *was seen as a hallmark of readiness for discharge from specialist care. Significant negative outcomes, including post transition "*loss to follow up" *or "*missed appointments*" were identified as negative outcomes associated with misaligned PCP-patient expectations and attitudes.

### Systems factors and transition of care

Features of the health care system, funding policies and existing processes of care were also seen to impact transition. The benefits of comprehensive communication between specialists and PCPs at discharge, and on an ongoing, urgent or as-needed basis after discharge were discussed at length in all focus groups. Utilization of an individualized plan of care and a structured discharge letter were identified as tools to achieve improved communication. It was suggested that this plan would support transitional care if it included most recent lab results, information about local resources and support services, current treatment regimens and targets, as well as specific advice for follow-up and next treatment steps. Including protocols for insulin therapy or other medication titration would also be useful. *"When I get [patients] back I would like to get instructions – if you run into this problem this is what you should do" (FG1) "...a flow sheet saying at our last visit this was the A1C, this was the blood sugar, this was the lipids with a little chart that would be a suggestion for how you could follow the patient... it would be the start of the transition ...and ultimately that same sheet could be photocopied and sent back when Mr. Smith runs into trouble..." (FG1)*.

Also of importance was individualized information about patient goals, barriers and facilitators to care, including patient preferences and readiness for participation in decision making. After discharge, ongoing, rapid access to all members of the multidisciplinary team by phone or email to "*get information back very quickly" *(FG1) was viewed as supportive as was confirmation that the PCP could re-refer if need be, *"...it would be nice to have an invitation back...from the endocrinologist at the end of that extensive letter" *(FG2).

The theme of the financial burden related to uninsured costs of diabetes medications, supplies and services was identified as a major barrier to transition for many individuals. The issue of reduced access to *"free services" *after discharge was repeatedly raised. Resources identified as being readily available in specialist centers but less accessible in primary care included the services of a dietitian, nurse educator, ophthalmologist, chiropodist, or psychologist. Inability to easily access or afford these services on an ongoing basis was viewed by some participants as a valid reason for continued care at a multidisciplinary specialist center. Additionally, maintenance of "*expensive polypharmacy" *initiated by specialists and the cost of blood glucose testing required as a result of intensive diabetes management were identified as transitional concerns "...*a real problem is the financial factor for the patients and realizing that when we ask them to record the sugar level and every strip costs a dollar...that's one thing the system should change" *(FG1).

## Discussion

The findings of this qualitative study build on the patient, provider and systems barriers previously identified in related literature [4,14]. While many primary care providers saw a clear role for diabetes specialist referral and are interested in having referred patients discharged back to their care, it was recognized that not all patients should be discharged from specialist care. In this study, we identified a range of factors at the PCP, patient and systems levels that can serve as barriers or facilitators to the discharge process. These three foci or broad categorization of barriers and facilitators to discharge are aligned with those identified in 2001 by Brown et al. in their investigation of barriers and facilitators to Ontario family physician's management of diabetes [4]. While these investigators were interested in identifying factors that would facilitate use of diabetes best practice guidelines, we have confirmed that more than six years after this publication, issues of time, physician remuneration, cost of diabetes services and medication, gaps in PCP knowledge and skill, as well as patient compliance remain factors associated with successful diabetes care in family practice.

While study participants appeared to have benefited from efforts of professional bodies, academic centers, governments and industry to disseminate diabetes management guidelines as evidenced by high levels of awareness of these guidelines, many PCPs were not confident about participating in insulin adjustment and facilitating patient behaviour change [14]. This PCP uncertainty related to providing behavioural support resonates with the findings of the international DAWN study, which identified that psychosocial support needs for those living with diabetes were less likely to be identified or addressed by generalists as compared to diabetes specialists [21]. Our study suggests that specialist centers can support PCP capacity to provide complex diabetes care by establishing interactive, applied learning opportunities such as clinic based learning, more formalized mentorship, and easy phone access for "just in time" specialist advice.

At the interface between patient and provider, transition may be supported when attitudes and expectations related to treatment options, outcomes, and patient self-management behaviours are aligned. While PCPs in our study identified development of patient self- determination and attitudinal change as desired goals of specialist referral, it was evident that they did not always feel comfortable with actually sharing responsibility for care when patients returned from specialists clinics with these new attitudes, behaviours and expectations of the care provider-patient relationship. Language used by several participants in this study suggests that some PCPs continued to view diabetes care according to a more traditional hierarchical, "compliance" paradigm while most multidisciplinary diabetes clinics now operate from a participatory, collaborative, "empowerment" perspective. Application of more authoritarian approaches to diabetes care have been associated with thwarted diabetes self management efforts, negative clinical outcomes, and patient loss to follow-up [22]. This is of particular concern given the growing younger population with diabetes who value more aggressive treatment and participative health care decision making [23].

Previously published literature in diabetes and other chronic illnesses reflects a growing interest in the association amongst patient- provider characteristics and relationships, chronic disease self-management, and clinical outcomes [6,22-25]. The impact of these factors on discharge from diabetes specialist care has not previously been well studied, however, our research indicates that it may be important to successful transitions. Developing and maintaining respectful, trusting relationships between specialist and PCPs can enhance patient confidence in the care they receive [9,25]. Specialist diabetes care centers can play a role in assisting PCPs to develop competence and confidence in utilizing approaches to diabetes care that support behavioural change, patient empowerment, and more participatory roles for patients who desire it.

Poor communication between caregivers has been associated with adverse patient outcomes and may be a reason why specialists are hesitant to discharge patients back to primary care or why primary care physicians may be reluctant to accept them back [15,26,27]. As identified in other, non diabetes specific studies, discharge is promoted when tools or strategies that support effective, timely communication are in place [11-13,15,28]. The suggestions of study participants to utilize transitional communications tools such as patient specific care plans, structured discharge letters, or detailed flow sheets are aligned with these previous findings.

Finally, system support for patients and providers can affect discharge from specialist care. Current fee for service reimbursement models for PCPs may not reflect the time required for complex diabetes management, behavioural support and counselling. Likewise, provision of specialist advice to PCPs by telephone or electronically is not remunerated. Developing systems for diabetes care that improve direct access to allied health professionals, and that include expanded roles for nurses and dietitians with diabetes expertise should be considered. Similarly, establishing health care policy that relieves the current burdens associated with purchasing diabetes medications and monitoring supplies would facilitate diabetes management in all settings.

### Study Limitations

This study is limited by its sampling approach. Participants were volunteers interested in participating in a discussion about discharge from specialist care. On average, they had more than 20 years of experience as practicing primary care providers and most worked in an urban setting. More recent graduates or rural practitioners may have different views regarding transitional care. The unique regional health delivery systems and policies may have influenced participants' responses; however, many findings of this study are aligned with literature published from other practice settings.

### Future Research

While this study provides new insight into perspectives of primary care providers related to the transition of diabetes care, further research is required to explore the experiences of a wider sample of practitioners and validate the applicability of themes identified in this exploratory study. It will also be important to gain a fuller understanding of the determinants of successful transitional care from the perspectives of patients and specialist team members.

Specialist diabetes referral often includes both optimization of pharmacological treatment and self-management education using an empowerment approach. The impact of referral on patient expectations of primary care providers in the context of a largely self-managed condition such as diabetes, was identified as a potential, but understudied contributor to successful transition.

Finally, there appears to be a need to develop and evaluate evidence based, patient centered tools to support improved discharge communication between specialists, patients and primary diabetes care providers.

## Conclusion

This exploratory study highlights the complex interdependencies associated with transitions in diabetes care. Given the growing demand for diabetes services, developing strategies that will assist with the timely, appropriate and supported transition of responsibility for diabetes care from specialists back to primary care may be one way of ensuring future access to specialist services for those with complex care needs.

This study underlines the breadth of PCP concerns related to transition of diabetes care from specialist clinics. Utilization of tools that promote timely information flow between care providers is encouraged, however, implementation of strategies that enhance PCP competencies and confidence related to diabetes management will be equally important to successful transitions. Developing funding policies that support the establishment of mentoring programs and applied learning opportunities for PCPs and that improve access to multidisciplinary diabetes specialist team members would also facilitate discharge from specialist programs. Understanding the concerns of all stakeholders, not only PCPs, but specialists and patients alike, will be necessary to develop strategies that support the transitions of individuals with complex diabetes needs across the continuum of care.

## Ethics approval

This research was reviewed by the Ottawa Hospital Research Institute Ethics Board and received approval on March 7, 2006. Protocol #2005434-01H.

## Competing interests

The authors declare that they have no competing interests.

## Authors' contributions

SB contributed to conception and design of the study, developed data collection tools, participated in data analysis and interpretation, drafted and revised the manuscript. MR contributed to conception and design of the study, developed data collection tools, participated in data collection, analysis and interpretation, and manuscript review. J M contributed to conception and design of the study, participated in data collection and manuscript review. SI contributed to conception and design, participated in recruitment, and manuscript review. J M provided study coordination, participated in data collection, and manuscript review. CL contributed to conception and design of the study, participated in recruitment, and manuscript review. EK contributed to design of the study, participated in recruitment and manuscript review. TCO contributed to conception and design of the study, and participated in manuscript review. All authors read and approved the final manuscript.

## Pre-publication history

The pre-publication history for this paper can be accessed here:


